# Animal Models of Rheumatoid Arthritis (I): Pristane-Induced Arthritis in the Rat

**DOI:** 10.1371/journal.pone.0155936

**Published:** 2016-05-26

**Authors:** Jonatan Tuncel, Sabrina Haag, Markus H. Hoffmann, Anthony C. Y. Yau, Malin Hultqvist, Peter Olofsson, Johan Bäcklund, Kutty Selva Nandakumar, Daniela Weidner, Anita Fischer, Anna Leichsenring, Franziska Lange, Claus Haase, Shemin Lu, Percio S. Gulko, Günter Steiner, Rikard Holmdahl

**Affiliations:** 1 Division of Medical Inflammation Research, Department of Medical Biochemistry and Biophysics, Karolinska Institutet, Stockholm, Sweden; 2 Department of Internal Medicine 3, University of Erlangen-Nuremberg, Erlangen, Germany; 3 Division of Rheumatology, Medical University of Vienna, Vienna, Austria; 4 Redoxis AB, Medicon Village, Lund, Sweden; 5 Fraunhofer Institute for Cell Therapy and Immunology, Leipzig, Germany; 6 Department of Immunopharmacology, Novo Nordisk A/S, Malov, Denmark; 7 Department of Biochemistry and Molecular Biology, School of Basic Medical Sciences, Xi'an Jiaotong University Health Science Center, Xi'an, Shaanxi, PR China; 8 Division of Rheumatology, Department of Medicine, Icahn School of Medicine at Mount Sinai, New York, New York, United States of America; Macau University of Science and Technology, MACAO

## Abstract

**Background:**

To facilitate the development of therapies for rheumatoid arthritis (RA), the Innovative Medicines Initiative BTCure has combined the experience from several laboratories worldwide to establish a series of protocols for different animal models of arthritis that reflect the pathogenesis of RA. Here, we describe chronic pristane-induced arthritis (PIA) model in DA rats, and provide detailed instructions to set up and evaluate the model and for reporting data.

**Methods:**

We optimized dose of pristane and immunization procedures and determined the effect of age, gender, and housing conditions. We further assessed cage-effects, reproducibility, and frequency of chronic arthritis, disease markers, and efficacy of standard and novel therapies.

**Results:**

Out of 271 rats, 99.6% developed arthritis after pristane-administration. Mean values for day of onset, day of maximum arthritis severity and maximum clinical scores were 11.8±2.0 days, 20.3±5.1 days and 34.2±11 points on a 60-point scale, respectively. The mean frequency of chronic arthritis was 86% but approached 100% in long-term experiments over 110 days. Pristane was arthritogenic even at 5 microliters dose but needed to be administrated intradermally to induce robust disease with minimal variation. The development of arthritis was age-dependent but independent of gender and whether the rats were housed in conventional or barrier facilities. PIA correlated well with weight loss and acute phase reactants, and was ameliorated by etanercept, dexamethasone, cyclosporine A and fingolimod treatment.

**Conclusions:**

PIA has high incidence and excellent reproducibility. The chronic relapsing-remitting disease and limited systemic manifestations make it more suitable than adjuvant arthritis for long-term studies of joint-inflammation and screening and validation of new therapeutics.

## Introduction

There is a critical need for better and well-defined animal models for rheumatoid arthritis (RA) that display the specific aspects of the human disease and can serve as platforms for research on the underlying pathology, as well as for drug discovery and validation [1,2]. Currently, collagen type II-induced arthritis (CIA) in mice and rats, and adjuvant arthritis (AA) in rats are the most widely used arthritis models in academia and industry [3–5]. AA, which is induced by a mixture of paraffin oils, mannide monooleate, and heat-killed mycobacteria (Mb), known as complete Freund′s adjuvant (CFA), is an acute model that tends to have an aggressive disease course [6]. Although the aetiology of AA remains unclear, a major immunogen in AA has been shown to be an HSP65-derived peptide from Mb [7] that triggers an autoreactive T cell response against proteins in the joints [8]. However, AA is not a joint-specific disease as it is accompanied by severe systemic manifestations, such as splenomegaly and hepatomegaly [9,10] that are atypical of RA.

Administration of paraffin oils and mannide monooleate can also elicit arthritis in the absence of Mb (i.e. incomplete Freund's adjuvant, IFA). This so-called oil-induced arthritis (OIA) model is acute and relatively mild compared to AA [11,12]. A minor component of IFA is a saturated 19-carbon alkane known as pristane (2,6,10,14-tetramethylpentadecane) [13], which induces chronic relapsing arthritis in DA rats when injected in pure form [14]. Similar to other rat adjuvant-models, initiation and perpetuation of pristane-induced arthritis (PIA) is dependent on CD4+ T cells [14,15]. However, in contrast to AA, self, rather than foreign, MHC class II-restricted antigens initiate the immune response in PIA. The specificities of these antigens remain largely unknown, although T cell recall responses have been demonstrated to both ubiquitous and joint-specific antigens in acute and chronic PIA [16,17]. In addition, through genetic mapping in inbred strains, we have recently demonstrated an association between PIA and certain alleles of RT1-B (the rat orthologue of HLA-DQ) [18]. Thus, the pre-clinical phase of PIA appears to reflect the early events of RA, involving a polyclonal expansion of MHC-II-restricted self-reactive T cells. Moreover, the onset of overt PIA is characterized by an increased acute phase response, which together with the symmetrical disease manifestations, presence of IgG rheumatoid factors, and the chronic disease course establishes PIA as one of the few models that fulfils the American College of Rheumatology (ACR) classification criteria for RA [16,19–21]. In addition, the chronic relapsing disease course and limited systemic disease manifestations make it a suitable model to study long-term effects of autoinflammatory processes relevant to RA. The current study provides a comprehensive characterization of the various factors that influence the disease course of PIA. The included protocol can further serve as a guide for performing experiments using this model.

## Materials and Methods

### Ethics statement

All rats used in this study were maintained and handled in strict accordance with the Swedish Animal Welfare Act. The protocol was approved by the Regional Ethical Committees for Animal Research in Stockholm, Sweden (permit ID; N67/10, M107-07) and by the European Community Council Directive (86/609/EEC). Rats were anesthetized in 3% isoflurane using oxygen as carrier gas and sacrificed using carbon dioxide.

### Animals

DA/OlaHsd founders originating from Harlan Laboratories (Harlan Europe, The Netherlands) were maintained in a barrier facility by sister-brother mating and were kept specific pathogen free (SPF) according to the current FELASA guidelines [22] (S1 Table contains a list of tested pathogens). Animals were housed in groups of 5 individuals per cage in climate-controlled (24°C/54±1% humidity) individually ventilated microisolator Type 4S cages (1400 cm^2^; Allentown Inc. Allentown, NJ, USA) containing aspen chips bedding material (Tapvei, Scanbur, Sollentuna, Sweden). Rats were fed autoclaved food (R70, Lantmännen, Sweden) and water *ad libitum*, and were subjected to 14 h light/ 10 h dark cycles [23]. Rats depicted Conv/Open were maintained in a conventional facility (12 h light/dark) with open cages. Conventional rats received the same diet and had the same bedding material as the barrier rats. Sentinel rats housed in the same conventional facility were positive for Pasteurella, Helicobacter spp. and pinworms (S1 Table). For certain experiments, rats were obtained from Harlan Laboratories, Charles River Laboratories or Janvier Labs.

### Arthritis induction and disease course definitions

PIA was induced in 8–11 week old rats by an intradermal injection of 100 μl pristane (2,6,10,14-tetramethylpentadecane, 95%, Acros Organics, Morris Plains, NJ, USA) at the dorsal side of the tail base if not stated otherwise. Adjuvant, oil-induced and collagen-induced arthritides were induced by intradermal injections of 100 μl IFA (Difco Laboratories, Detroit, MI, USA) containing 0.4 mg of *Mycobacterium butyricum* (Difco), 300 μl pure IFA, and 0.3 mg pepsin-digested collagen type II (CII) purified from rat chondrosarcoma [24], dissolved in 150 μl 0.1 M acetic acid and emulsified in an equal volume of IFA, respectively. Synthetic pristane was obtained from Sigma-Aldrich (P2870; St. Louis, MO, USA). Treated and non-treated rats or rats subjected to different immunization protocols were housed together in cages. The evaluation of clinical arthritis is described in detail in S1 Text. In brief, 1 point was given for each inflamed knuckle or toe and up to 5 points was given for an affected ankle (in total 15 points per paw, 60 points per rat). Scores were not given for deformations if not accompanied by erythema. The day of disease remission is defined here as the first of at least three consecutive scoring days with declining arthritis scores. The 'first relapse' (Table 1) is the first of at least three consecutive scoring days with increasing scores following a period of disease remission. The frequency of chronic arthritis is defined as the proportion of rats with a mean score of ≥5 or at least two days with scores >6 following day 60 after immunization.

**Table 1:** Incidence, onset, max severity and frequency of chronic arthritis in DA rats with PIA.

						Acute PIA						Chronic PIA		
										Weight Weight				
Exp	n	Sx	Age*	Month^†^	Dur.^‡^	%	Onset^§^	Max Day^¶^	Max sc.^|^	Max %**	Dis. corr.^††^	%^‡‡^	1^st^ Rlps^§§^	Max sc.^¶¶^
1	40	M	12	May	240	100	11.9±1.7	20.3±4.0	36.9±8.5	-19.4±4.3	0.55 (.0003)	100	85.2±22	27.2±9.2
2	20	M	9–10	March	127	100	11.3±2.2	23.7±4.0	39.7±11	-15.2±5.6	0.84 (.0001)	90	78.2±16	19.9±8.9
3	9	M	12	Dec	117	100	11.4±1.4	19.4±3.3	38.1±6.6	-18.3±2.3	0.14 (n.s)	100	77.4±18	27.7±5.1
4	16	M	9–10	Feb	109	100	11.7±1.5	23.1±3.2	30.1±10	-14.0±4.5	0.70 (.003)	63	74.6±10	15.2±7.0
5	24	M	7–12	Dec	100	100	10.3±1.8	19.4±4.6	36.4±10	-6.5±3.8	0.70 (.0001)	92	70.7±12	31.0±13
6	25	M	14–20	Dec	100	100	13.5±1.9	22.8±5.0	34.9±13	-5.4±4.2	0.62 (.001)	92	73.1±12	28.5±9.6
7	20	F	11–12	April	83	100	11.4±1.6	20.0±4.8	34.4±11	-12.2±4.0	0.66 (.002)	56	73.4±7.0	21.0±6.2
8	15	F	11–12	Nov	22	100	12.0±1.5	19.3±2.2	26.2±10	-12.7±4.7	0.54 (.038)	n.d	n.d	n.d
9	29	M	11–17	Oct	88	100	13.5±2.3	24.2±9.3	33.6±13	n.d	n.d	93	71.0±10	26.4±10
10	30	M	7–8	Jan	100	100	10.3±1.4	18.4±2.9	34.7±11	n.d	n.d	90	68.2±13	25.3±13
11	20	F	9–15	July	18	95	12.3±2.1	16.6±1.0	29.4±10	n.d	n.d	n.d	n.d	n.d
12	23	M	7–11	July	18	100	11.3±1.6	16.8±1.3	32.6±14	n.d	n.d	n.d	n.d	n.d

* Age (in weeks) of animals at the start of the experiment;

^†^ Month in which the experiment was started;

^‡^ Duration of the experiment (in days);

^§^ Mean day of onset (±SD);

^¶^ Mean day of max score (±SD);

^|^ Mean maximum score (±SD);

** Mean maximum weight loss compared to weight at onset (except for experiments 5 and 6, which show weight loss at day 17 vs day 13);

^††^ Correlation (r) between maximum score and maximum weight loss. Values in parentheses depict P-value (n.s. = not significant);

^‡‡^ Frequency of rats with chronic arthritis (see Methods for definition);

^§§^ Mean day of first relapse;

^¶¶^ Mean maximum score at chronic phase (arthritic animals only);

n.d. = not determined.

### Imaging

For histological comparison between late stage PIA and OIA, paws from rats 130 days after injection of pristane or IFA, respectively, were collected and decalcified with EDTA. Serial paraffin-embedded tissue sections of hind paws were analyzed for identification of osteoclasts by tartrate-resistant acid phosphatase (TRAP) staining (leukocyte acid phosphatase kit, Sigma-Aldrich). Microcomputed tomography of paws was performed using the cone-beam Desktop Micro Computer Tomograph “μCT 40” by SCANCO Medical AG, Bruettisellen, Switzerland. The settings were optimized for calcified tissue visualization at 45 kVp, 177 μA, and 250 ms integration time. For the segmentation of 3D-volumes, an isotropic voxel size of 9,7 μm and an evaluation script with adjusted grey-scale thresholds of the operating system “Open VMS” by SCANCO was used.

### Disease markers

Alpha-1 acid glycoprotein (AGP) was measured in serum diluted 1:20,000 by ELISA according to manufacturer’s instructions (Life Diagnostics, West Chester, PA, USA). Concentration of blood leukocytes was measured on a Sysmex KX-21N cell counter. The frequency of neutrophils was determined by flow cytometry (BD LSR-II) after staining with a monoclonal antibody against granulocytes (clone His-48, BD Pharmingen) or according to scatter profile. The results were analyzed with FlowJo (Tree Star Inc., Ashland, OR). Anti-CCP was measured with the Immunoscan CCPlus Kit RA-96PLUS, E-23-0182-07, Eurodiagnostica. Serum was diluted 1:25 in dilution buffer and plates were washed with the supplied wash solution. Positive, negative and reference control samples included in the Kit were used as controls. IgG were detected with 100 μl/well HRP Goat anti-rat IgG (clone: Poly4054) Cat. 405405 (Lot: B140787) Biolegend or HRP Anti-Human IgG Cat. 555788 (Lot: 49264) BD were used for detection of the human reference sample. ELISA plates were developed using TMB substrate solution. Incubation and wash steps were performed according to the manufacture’s instructions. Absorbance was read with the Synergy 2 microplate reader (BioTek Instruments, Inc.).

### Treatments

Etanercept (Enbrel; Immunex, Thousand Oaks, CA, USA) was administrated s.c. in 0.2 ml PBS (12.5 mg/ml) or i.v. (0.2 ml, 6 mg/ml). Fingolimod (Cayman Chemical Company, Ann Arbor, MI, USA; cat number: 10006292; lot: 0426940–36) was dissolved at 0.8 mg/ml in 100% ethanol, diluted to 0.4 mg/ml in PBS and administrated s.c. or orally at 1 mg/kg body weight. Control rats received an equal volume of ethanol (50%)/PBS vehicle. Cyclosporine A (Sandimmune, Novartis, Sweden) was administrated i.p. at 10 mg/kg. Methotrexate (Sandoz, Denmark) was administrated i.p. at 0.1 mg/kg or 0.05 mg/kg. Dexamethasone (Sigma Aldrich; cat number: D4902) was reconstituted in 70% ethanol, diluted in PBS to 0.32 mg/ml, and administrated i.p. at 1 mg/kg.

### Statistical analyses

Two group comparisons of non-normally distributed data (arthritis scores, weight change, day of disease onset and day of maximum arthritis) were performed using the Mann-Whitney U test. For comparison of more than two groups, the reported P-values were corrected for multiple comparisons using the Kruskal-Wallis test with a Dunn post hoc test. Weight change was calculated as percent weight-loss or weight-gain compared to disease onset. Linear regression was performed to analyze the correlation between acute and chronic arthritis, as well as between arthritis scores and other disease parameters (weight change, acute phase reactants and blood neutrophil counts). The coefficient of variation (CV) was used to determine the distribution in arthritis scores between immunization groups or, for the assessment of cage effects, individual cages, and is expressed as percent of the mean score. In all experiments a *P*-value less than 0.05 was considered significant. All statistical analyses were done with GraphPad Prism version 5 (GraphPad Prism software Inc. San Diego, CA).

## Results

### Pristane-induced arthritis is a chronic-relapsing model with high incidence and reproducibility

To best illustrate the various clinical aspects of PIA, we compared the disease course between rats immunized with pristane (PIA), IFA (OIA), IFA supplemented with *Mycobacterium butyricum* (AA), and rat CII emulsified in IFA (CIA).

**Fig 1:**
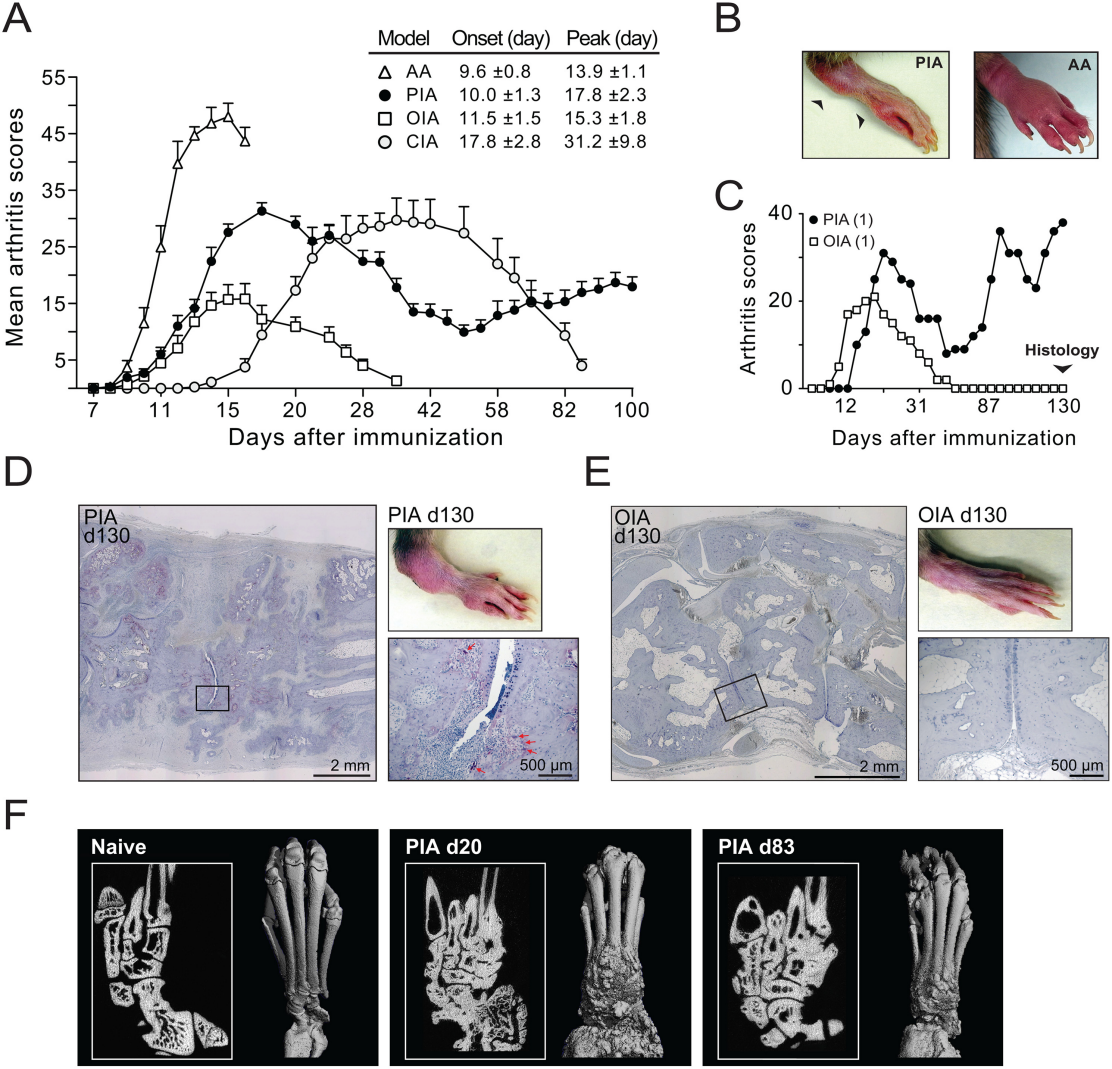
Disease development in different rat models of arthritis. (A) Arthritis scores of DA rats with pristane-induced arthritis (PIA), oil-induced arthritis (OIA), adjuvant arthritis (AA) and collagen-induced arthritis (CIA). The inset table shows day of onset (±SD) and day of maximum arthritis (Peak). The data are from four different experiments but were evaluated by the same investigator; all experiments except OIA were performed with male rats; AA (n = 7), PIA (n = 33), OIA (n = 15), CIA (n = 22). Error bars represent S.E.M. (B) *Left*: Hind paw from a rat with PIA showing arthritis in the ankle and MCP joints (arrows). *Right*: Hind paw from a rat with AA showing the characteristic whole-paw edema. (C) Arthritis in rats injected with pristane (PIA) or IFA (OIA). Data show total scores per rat. (D-E) TRAP stainings of hind paws of rats shown in (C) reveal ongoing resorption of cortical bone by osteoclasts (red arrows), inflammatory cell infiltrates, and extensive formation of new bone on day 130 after injection of pristane (D). In contrast, paws 130 days after IFA injection show inconspicuous bone and joint morphology (E). (F) Microcomputed tomography slice images (insets) and three-dimensional surface rendering of the tarsal bones of naive rats and rats 20 and 83 days after pristane-injection Massive new bone formation and ankylosis of adjacent bones can be seen in both acute and chronic PIA (images were acquired post-mortem and are therefore from different animals).

The T cell-driven adjuvant models, PIA, OIA and AA, demonstrated an earlier and more synchronized disease onset compared to the antibody-mediated CIA model (Fig 1A). Also the progression to severe arthritis was faster in these models; AA, OIA and PIA peaked within 4, 5 and 8 days of onset, respectively, whereas CIA peaked first after 13 days (*P* = 0.05 vs. PIA). Similar to other adjuvant models [25], the first paws affected in PIA were the hind paws, either alone (44% of 197 rats analysed) or together with the front paws (48%). The first joints affected were often the larger joints of the ankle or the metacarpophalangeal joints of the knuckles (Fig 1B, left). Massive edema affecting the entire paw, which is common in CIA [5] and AA (Fig 1B, right), was less frequent in PIA.

Disease remission in the adjuvant models usually begins 15–25 days after immunization; however, while rats with AA [12] and OIA (Fig 1A) recover from arthritis, the majority of rats with PIA developed a chronic relapsing-remitting disease course (Fig 1A and 1C and Table 1). Histological examination of hind paws at day 130 after disease induction revealed ongoing inflammation and accumulation of multinucleated TRAP+ osteoclasts at sites of erosion in joints from rats with PIA (Fig 1D). By contrast, previously affected paws from rats with OIA were completely healed at day 130 after immunization (Fig 1E). In microcomputed tomography images, massive new bone formation is the most characteristic feature of PIA (Fig 1F). Bony outgrowths and ankyloses accompanied by continuous destructive processes are apparent already in early stages of the disease (Fig 1F).

To assess the reproducibility of PIA, and determine the average incidence, day of onset, disease severity, and frequency of rats with chronic arthritis, we compared data across 12 experiments performed over five years in one facility using a total of 271 rats (Table 1). The results confirm the high incidence of arthritis in rats injected with pristane [14]. In addition, we found extremely small inter-experimental differences in disease onset, mean day of maximum arthritis and severity. The arthritis severity in the acute phase correlated significantly with the severity present in the chronic phase in 4 out of 9 chronic experiments (*r* = 0.42–0.62; *P* = 0.02–0.0003). The mean frequency of chronic arthritis for the experiments shown in Table 1 was ~86% but approached 100% in experiments that lasted >110 days, suggesting that all rats might eventually develop chronic disease, but that the time span until the first relapse varies between individual rats.

Taken together, PIA is an extremely robust, chronic-relapsing model with high incidence and moderate severity compared to the AA model.

### Optimization of dosing and immunization procedures

The induction of AA and CIA is most efficient via the intradermal (i.d.) route [6,26], which can possibly be explained by the fact that exogenous antigens are more efficiently taken up and processed by dermal dendritic cells [27]. In PIA, no exogenous antigens are administrated and hence the route of immunization may play a less important role.

**Fig 2:**
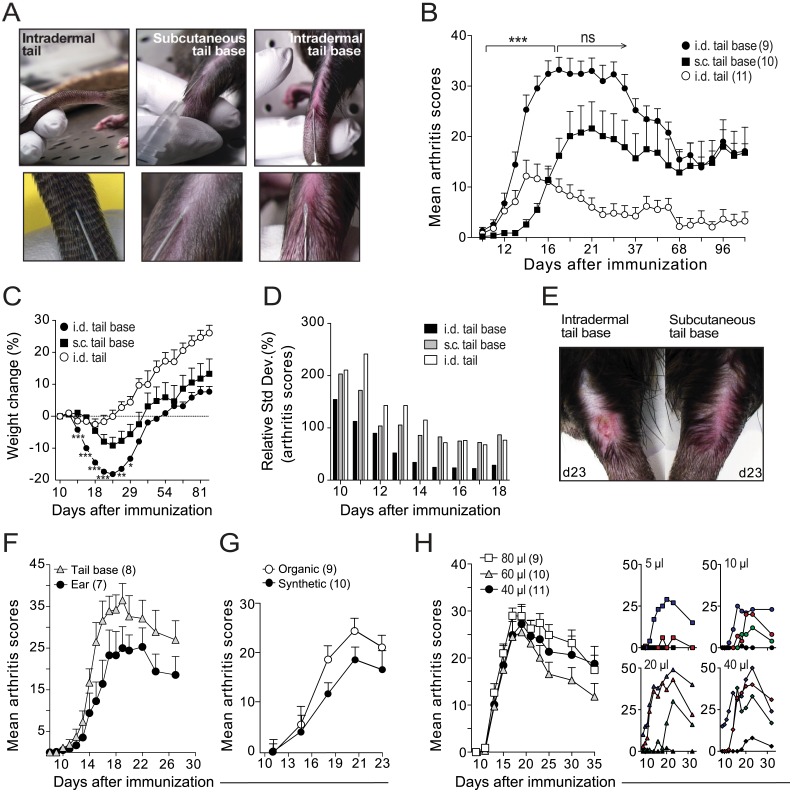
Intradermal (i.d.) immunization at the base of the tail induces a more severe arthritis with an earlier onset and less variation compared to subcutaneous (s.c.) immunization. (A) Male rats were injected i.d. midway between the tip and the base of the tail (left), s.c. (centre), or i.d. (right) at the base of the tail. Note that in contrast to s.c. immunization, the bevel of the needle is clearly visible through the skin when performing an intradermal injection as shown in the right picture. (B) Arthritis scores of rats immunized as in (A). Numbers in brackets indicate number of rats per group; statistical differences were only determined between groups injected at the tail base. (C) Weight changes compared to day 10 for rats shown in (B). Statistical differences determined between tail base injected rats. (D) Variation in arthritis scores between immunization groups (determined as coefficient of variation, in percent). Low values represent small variations. (E) Representative photographs of the injection site on day 23 after immunization. (F) Development of arthritis after injection of pristane i.d. at the base of the tail or in the pinna of the ear. (G) Arthritis in rats injected with pristane from shark-liver oil (organic) or from a synthetic source. (H) Disease development after i.d. injection of different doses of pristane. Right figures show arthritis development of individual rats. Statistical differences were determined by Mann-Whitney; *, P<0.05; **, P<0.01; ***, P<0.001.

To assess this, we injected rats with 100 μl pristane (1) i.d. at the base of the tail; (2) i.d. at mid-tail; or (3) subcutaneously (s.c.) at the base of the tail (Fig 2A). Rats from these three groups were cage-mixed and independently evaluated for arthritis by two investigators. We found that rats in group 1 developed more severe arthritis (Fig 2B and 2C) and exhibited less variation in arthritis scores (Fig 2D) compared to rats in groups 2 and 3. Interestingly, the two groups injected intradermally developed arthritis significantly earlier than rats injected s.c. despite showing very different severity patterns. The early onset in rats injected i.d. suggests that dermal DCs could be critical for the priming of arthritogenic T cells in PIA despite the absence of exogenous antigens in this model.

Severe lesions at the injection site are frequently seen in animals injected with adjuvants containing Mb [28] but were not observed in rats with PIA. However, injection of pristane i.d. resulted in erythema at the injection site, and a few individuals developed lesions of the kind shown in Fig 2E. These lesions did not bleed or penetrate through the subcutaneous layer of the skin, however, and they resolved spontaneously after 3–4 weeks (data not shown).

Pristane injected at the base of the tail drains directly to the inguinal and axillary lymph nodes [29]. To specifically target these lymph nodes did not seem to be critical for the development of arthritis, however, as injection of pristane into ear pinna, which targets the cervical lymph nodes in the neck, induced disease with similar severity and involvement of joints (Fig 2F and data not shown).

**Fig 3:**
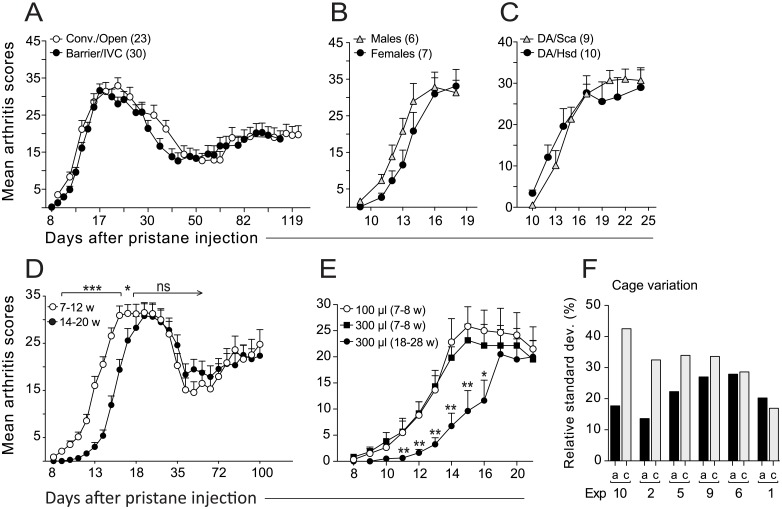
PIA is dependent on age but not gender, housing conditions and environment. (A) The development of PIA in rats bred in a conventional facility with open cages (Conv./Open) or a barrier facility with IVC cages (Barrier/IVC). The two groups differed in pathogen status (see Methods). (B) Female and male DA rats were equally susceptible to PIA (see also Table 1). (C) DA rats from Charles River (Sca) and Harlan (Hsd) showed similar arthritis development. (D) PIA in 7–12 (n = 24) and 14–20 (n = 25) week old male rats; statistical differences were determined as in Fig 2. (E) Increasing the dose of pristane did not accelerate the onset of arthritis in older rats (7–8 rats/group). Statistical differences were determined between groups injected with 300 μl pristane. (F) Cage-effects in acute (a) and chronic (c) arthritis. Bars depict the variation in mean maximum scores between cages (determined as coefficient of variation, in percent). Experiment (Exp) numbers refer to numbers in Table 1.

We further evaluated pristane from different commercial sources and found that synthetic pristane was equally arthritogenic as pristane isolated from shark-liver oil (Fig 2G). Moreover, we found that 40 μl pristane induced the same severe arthritis as 80 μl (Fig 2H) and there was no difference between 100 μl and 300 μl (Fig 3E). In fact, we observed severe arthritis with as small doses as 5 μl pristane (Fig 2H).

In summary, administration of pristane i.d. induced more severe arthritis with less variation compared to s.c. immunization. Pristane from synthetic and organic sources were equally efficient in inducing arthritis and we did not see a correlation between arthritis severity and the amount of pristane administrated between injection volumes from 40 to 300 μl. Lower doses, however, decreased incidence of PIA.

### The susceptibility to PIA is age-dependent but independent of gender and housing/environmental conditions

Raising animals under germ-free conditions may either attenuate [30] or enhance their susceptibility to arthritis [31,32]. Although it remains to be investigated how changes in microbiota influence the susceptibility to PIA, here, we compared the development of arthritis in rats from a conventional facility with open cages to rats in a barrier facility with IVC cages (Fig 3A). Despite the different pathogen statuses between these facilities (*see* Methods), we did not observe any differences in the development of acute and chronic PIA between the two groups, suggesting that there are limited infectious organism and environmental constraints on this model. We further compared arthritis development between males and females, and between DA rats from two different vendors (Harlan and Charles River). We found no differences in the susceptibility to PIA between genders (Fig 3B and Table 1), or between the two colonies (Fig 3C). By contrast, age played an important role for the arthritis development: younger rats demonstrated an earlier onset but similar progression, severity and development of chronic arthritis as older rats (Fig 3D and Table 1). Importantly, increasing the dose of pristane to 300 μl to adjust for body weight did not accelerate the onset of PIA in the older rats (Fig 3E).

Social interactions between animals may cause variation in disease severity across cages. We compared the mean scores between cages at the peak of the acute and chronic phases of PIA and determined the percentage of variance that can be explained by such “cage effects”. As shown in Fig 3F, the variation in mean maximum scores between cages was greater in chronic compared to acute PIA but was still much smaller than the variation observed in rats injected with pristane s.c. (Fig 2D).

Taken together, caging, gender, pathogen status and environment had little or no effect on PIA, whereas the age of the animals influenced the onset of the disease.

### Disease markers and treatment

Rats immunized with pristane showed normal weight gain before the onset of clinical disease (data not shown). The decline in body weight that followed on the onset of arthritis was proportional to the disease severity and, hence, can be used as measure of disease activity (Table 1 and Fig 2C). Importantly, the weight-loss was only temporary: rats that lost 10–20% of their body-weight during the acute phase of PIA (Table 1), regained weight during disease remission (Fig 2C), suggesting that this weight-loss is probably not a consequence of cachexia.

**Fig 4:**
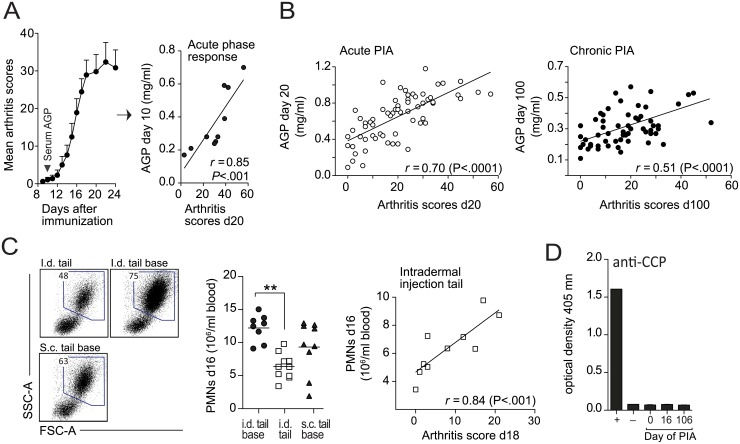
Disease markers and treatment. (A) Correlation between arthritis scores on day 20 and serum levels of alpha-1-acid glycoprotein (AGP) on day 10. (B) AGP-disease correlations on day 20 and 100 after immunization. (C) Representative FSC-SSC profiles of blood-leucocytes on day 16 post immunization of animals shown in Fig 2B. Numbers in gates represent percentage of live cells. Adjacent scatter plots show absolute numbers of polymorphonuclear cells (PMNs) on day 16 (P-value has been corrected for multiple comparisons) and correlation with arthritis scores on day 18. (D) Rats with PIA did not develop antibodies to cyclic citrullinated peptides (CCP). Plus (+), minus (-) and numbers below the graph represent controls and day after immunization (n = 30 per time-point), respectively (see Methods).

High levels of alpha-1-acid glycoprotein (AGP) at the onset of PIA correlated with the development of severe arthritis (Fig 4A). The levels of AGP continue to increase during the first 20 days of PIA and then decline rapidly [20]. However, despite the overall lower levels in chronic PIA, AGP was still significantly correlating with chronic disease activity (Fig 4B).

Similar to AGP, neutrophilia correlated strongly with the disease activity in PIA. We compared the absolute levels of polymorphonuclear cells (PMNs) in the blood at day 16 after immunization in rats injected with pristane i.d. or s.c. (shown in Fig 2A). Rats injected i.d. at the base of the tail, which developed the most severe form of disease, showed higher relative and absolute numbers of PMNs in the blood (Fig 4C). We also determined the presence of anti-citrullinated protein antibodies (ACPA) in acute and chronic PIA. ACPAs are highly associated with RA [33] but do not occur in most animal models of the disease [34], and were also absent in PIA (Fig 4D).

**Fig 5:**
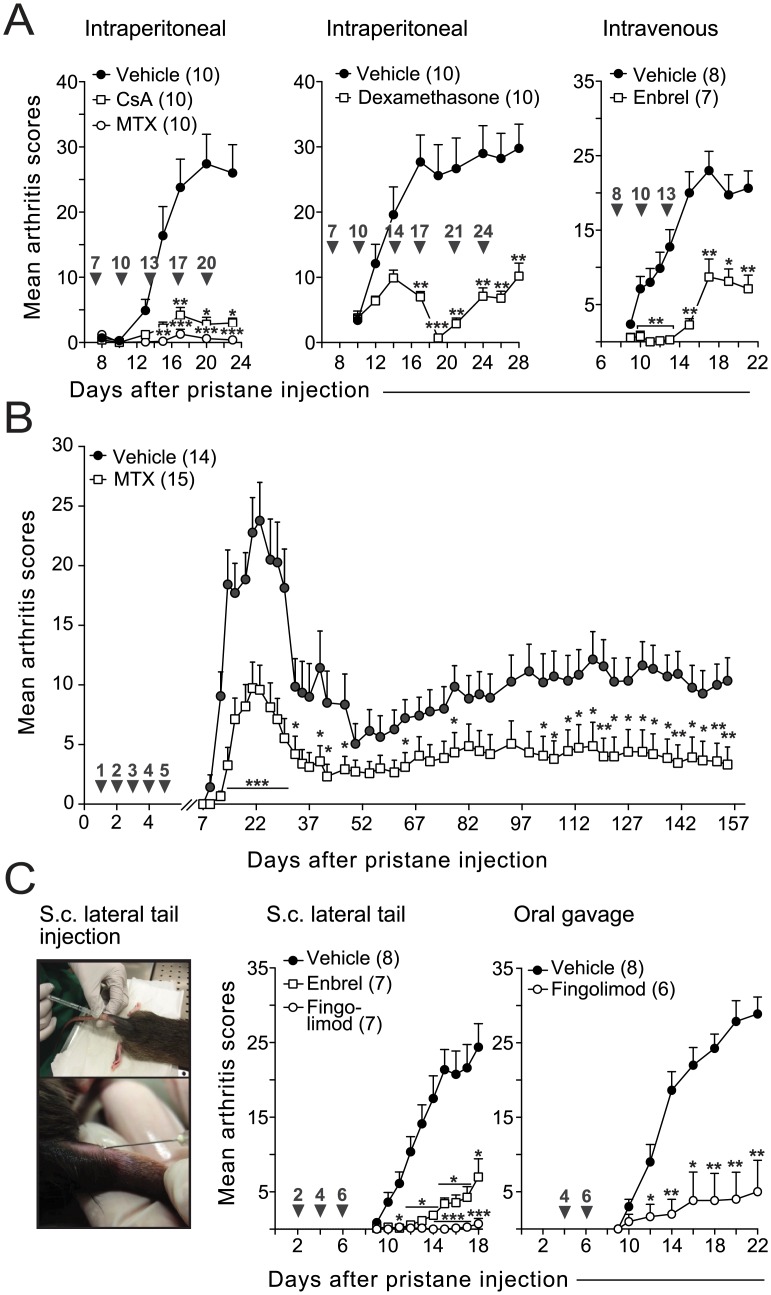
PIA serves as a suitable model to evaluate novel treatment strategies. (A) Treatment with Methotrexate (MTX), Cyclosporine A (CsA), dexamethasone and etanercept (Enbrel) in rats with early-established arthritis. (B) Early treatment with MTX ameliorates both acute and chronic arthritis. (C) Left photos show subcutaneous (s.c.) administration of Fingolimod (FTY720) in the flanks of the tail base. Note that the injection sites are clearly separated from the site of immunization (see Fig 2A). The right graphs show PIA in rats treated subcutaneously or orally with etanercept or fingolimod. Statistical differences were determined as in Fig 2. P-values in (A, MTX vs. CsA vs. vehicle) and (C, Enbrel vs. Fingolimod vs. vehicle) have been corrected for multiple comparisons. Black arrowheads indicate treatment days.

The standard anti-rheumatic drug methotrexate (MTX) has been shown to ameliorate inflammation in PIA [35]. Here we compared the administration of MTX to the TNF-inhibitor etanercept (Enbrel) and to two anti-inflammatory drugs: cyclosporine A (CsA) and dexamethasone. As demonstrated in Fig 5, these drugs efficiently suppressed acute (Fig 5A) and chronic arthritis (only tested for MTX, Fig 5B) when administrated i.v. or i.p. to rats with early established PIA or before onset of arthritis, respectively. To assess the usefulness of PIA as a model for validating new therapeutic candidates in RA, we further compared the efficacy of etanercept to fingolimod (Gilenya), a drug that was recently approved for oral treatment in multiple sclerosis and which inhibits transmigration of primed lymphocytes from the lymphoid organs to peripheral blood [36]. As shown in Fig 5C, fingolimod was at least as efficient as etanercept in reducing arthritis when delivered to pre-arthritic rats subcutaneously, and was also efficient when administrated orally (Fig 5C, right panel).

## Discussion

PIA is a highly reproducible animal model of RA with an incidence close to 100% and a high frequency of chronic arthritis. This model shows low sensitivity to variations in housing conditions and environment and therefore performs equally well between different research laboratories, which allows direct comparison between experiments and reproduction of results. In contrast to the more widely used AA model, the induction of PIA relies on a highly defined hydrocarbon instead of Mb antigens, which results in less local and systemic manifestations and therefore probably less discomfort for the animals.

Based on the data in the present and previous studies [14,17,37,38], the 1^st^ BTCure Animal Workshop in 2012 resulted in a set of general recommendations for the induction, evaluation and reporting of PIA (summarized in Table 2). The results presented here were mainly generated using the DA/OlaHsd strain from Harlan Laboratories, but we have also had good experience of using other substrains, including DA/Sca (Fig 3C), DA/HanRj (Janvier Labs) (Fig 5B) and DA/BklArbNsi (Bentin and Kingman) [39]. However, not all substrains of DA are equally susceptible to PIA [40] and genetic differences should be considered if animals are purchased from different vendors. The genetic control of the model is also likely to improve with the recently obtained genome sequence data of several inbred DA strains [41,42].

**Table 2:** Guidelines and recommendations for pristane-induced arthritis (PIA).

Step	Method/Description*	Comment	Reference
**Disease Induction**	Place anesthetized rat on its ventral side and straight-ten tail.	Anesthesia: Isoflurane (1–3%) and oxygen (2–3 dm^3^/min).	
	Wet fur at tail base with 70% ethanol and part hair along midline.	Insert needle with bevel facing upwards. The bevel should be clearly visible through the skin.	Fig 2A (right photo)
	Inject 100 or 150 μl synthetic pristane strictly intra-dermally. Withdraw needle slowly to avoid leakage.	A subcutaneous injection will increase variation in arthritis scores.	Fig 2D
**Evaluation**	Weigh rats on first scoring day and thereafter 2 times per week.	Disease onset is age-dependent. See Table 1 for expected day of onset.	Table 1
	Each paw can receive a max. score of 15: 5 p for a fully affected ankle and 1 p for each digit and knuckle.	Scores are not given for chronic deformations not accompanied by erythema.	S1 Fig
**Reporting**	Mean weight change (in %) and arthritis scores (both with SEM) should be depicted graphically.	Number of animals, age-range and substrain (including vendor) should be reported.	
	State caging conditions (mixed or separate groups).	Reporting should follow the ARRIVE guidelines.	[54]

* A detailed overview of each step is given in S1 Text.

The local environment and pathogen status of the animals were not critical for the development of PIA as shown by experiments in conventional and barrier facilities. Similar observations have also been made in other models in DA rats [43], suggesting that the low environmental constraints on this model are related to the high responsiveness of the strain. This may also explain why we did not observe a gender bias in the DA rats as previously been described in the less arthritis susceptible LEW strain [14].

Similar to other adjuvant models of arthritis, the onset of PIA is highly synchronized between individuals. Compared to AA, the disease course in PIA is less aggressive and induces a milder and only transient weight-loss, which, in contrast to AA [44], does not appear to be associated with cachexia. The model is therefore highly suitable for both acute and chronic therapeutic interventions and can be more easily accepted when animal welfare is taken into account. The onset of arthritis is affected by the age of the rats and the route of immunization. Younger rats developed arthritis earlier than older rats and i.d. compared to s.c. administration of pristane induced a more robust disease with an earlier onset and less variation in arthritis scores. Thus, it is recommended to inject i.d. to reduce the number of animals necessary for detecting significant effects between treatment groups or the like.

In contrast to the route of administration, in a range of 40–300 μl the amount of pristane was not critical for the incidence, onset, or severity of the disease. Severe arthritis could be induced by doses as low as 5 μl, albeit at a reduced incidence. We provide clinical and histological data showing that injection of 100 μl is associated with chronic joint damage. It is important to emphasize, however, that no dose-response experiments based on both clinical and histological data has been done in PIA, and that the most widely used dose in the literature is 150 μl of organic pristane. Organic pristane is produced by phytoplankton and accumulates naturally in the liver of animals, particularly in sharks [45,46]. Since several shark species are endangered and the organically produced oil may contain impurities that affect the development of arthritis, we recommend the use of 100 μl or 150 μl of synthetic pristane as a standard regimen for the induction of PIA.

Variation in arthritis scores between cages increased in the chronic phase of PIA but was still lower than in rats immunized with pristane s.c. Nevertheless, it is strongly recommended that any experiment, which compares treatment or genotype, is performed with mixed cages to avoid subjective bias. If the treatment regimen prevents the use of mixed cages, the cage will be the experimental unit and this should be stated in the study design. The disease evaluation should be performed in a blinded manner for the same reason. We recommend using the 60 [24] or 80-point [47] scoring systems for PIA, which both have been carefully evaluated by histology and shown to reflect the histological changes that takes place in the arthritic joint, including cartilage and bone erosions, synovial hyperplasia, and synovial inflammation [16,48]. Weight change can be used as an objective measure for the disease activity in PIA. In addition, AGP-levels and neutrophil counts in the blood correlate strongly with the disease persistence and progression. With regards to pro-inflammatory cytokines, IL-1β correlates with and partially precedes the course of clinical arthritis, whereas serum IL-6 and TNFα are not changed during PIA [49]. It is important to consider, however, that any disease intervention could have a direct separate effect on weight, acute phase response, or neutrophil levels. Similar, the effect on these parameters may depend on the specific health status of the rats.

Animal models are valuable tools for the study of chronic inflammatory diseases, such as RA. However, the model of choice needs careful consideration in regards to the specific research question addressed, since a single animal model cannot reflect every aspect of a human syndrome. To which extent then does the acute and chronic manifestations in PIA reflect human RA? The typical disease course in RA involves an erosive non-reversible destruction of joints, which eventually leads to cartilage damage [50] and periarticular bone loss [51]. Anabolic changes in the bone, such as the emergence of osteophytes, are also seen in RA although they are more prominent in other rheumatic diseases, in particular in psoriatic arthritis and ankylosing spondylitis [52]. For reasons still unclear, these anabolic changes are also fairly prominent in rodent models of arthritis [53], which may reflect and intrinsic type of responsiveness of cells residing in the rodent entheses that appear to proliferate and then calcify in response to surrounding inflammation. These bony outgrowths were also prominent manifestations in acute and chronic PIA (Fig 1F), as was multiple erosive lesions, surrounded by osteoclasts (Fig 1D), which are hallmarks of RA. Hence, there are distinct similarities as well as differences between a typical arthritic joint in PIA and RA.

Adjuvant models in rats complement the classical antigen-induced arthritis models in mice and fulfil an important role for drug screening and testing of new therapeutics in RA. We have shown that PIA offers distinct advantages over AA as a unique model of chronic arthritis for studying autoimmune joint inflammation in rodents.

## Supporting Information

S1 Fig Visual disease evaluation using the 60-pt scoring system. (A-B) For the visual inspection of the paws, rats can be placed in a standing position on a surface as demonstrated in (A) or grasped by hand as shown in (B). Note how the rat's right front limb is fixed between the index and middle fingers and how the head is supported by the grip around the neck. It is important that the grip is firm but not too hard. (C-E) According to the 60 pt scoring regime, a fully affected ankle should be given a score of 5, whereas an inflamed metacarpophalangeal joint or finger/toe (including inflammation in the proximal interphalangeal and/or distal interphalangeal joint) receive a score of 1 pt each. If the ankle is only partially affected (as in E) the score should be proportional to the inflamed area. (F) Schematic illustration of a hind paw. The numbers represent the maximum scores of each joint(s). (G) Deformation following acute arthritis is common in PIA but should not be given points if not accompanied by erythema.
(EPS)

S1 Table Health status of rats in barrier and conventional facilities. (DOCX)

S1 Text BeTheCure Guidelines for Pristane-Induced Arthritis. (DOCX)
