# Supplementary material for: Animal Models of Rheumatoid Arthritis (I): Pristane-Induced Arthritis in the Rat
Source: PLoS One. 2016 May 26;11(5):e0155936. doi: 10.1371/journal.pone.0155936 (PMC4881957; doi:10.1371/journal.pone.0155936)
Supplement: S1 Table — (DOCX) [file pone.0155936.s002.docx]

**S1 Table:** Health status of rats in barrier and conventional facilities.

| **VIRAL INFECTIONS (SEROLOGY)** | **Test Method** ^*^ | **Barrier** ^†^ | | **Conventional** ^‡^ |
| --- | --- | --- | --- | --- |
| Parvoviruses: | IF |  | |  |
| Kilham rat virus |  | - | | - |
| Toolan’s H-1 virus |  | - | | - |
| Rat parvovirus |  | - | | - |
| Pneumonia virus of mice | IF | - | | - |
| Sendai virus | ELISA | - | | - |
| Sialodacryoadenitits/Rat corona virus | ELISA | - | | - |
| Hantaan virus | IF | - | | - |
| Mouse adenovirus | IF | - | | - |
| Reovirus type 3 | IF | - | | - |
| **BACTERIAL INFECTIONS** |  | | | |
| Bordetella bronchiseptica | Cultivation | - | | - |
| Clostridium piliforme | IF | - | | - |
| Corynebacterium kutscheri | Cultivation | - | | - |
| Mycoplasma pulmonis | ELISA | - | | - |
| Pasteurellaceae | Cultivation | - | | X |
| Salmonella spp | Cultivation | - | | - |
| Streptobacillus moniliformis | Cultivation | - | | - |
| Streptococci-haemolytic | Cultivation | - | | - |
| Streptococcus pneumoniae | Cultivation | - | | - |
| Helicobacter spp | PCR | - | | X |
| **PARASITE INFECTIONS** |  | | | |
| Ectoparasites | Stereo-microscopy | - | | - |
| Endoparasites | Flotation/  Microscopy | - | | X ^4^ |
| **PATHOLOGICAL EXAMINATION** |  | |  | |
| Macroscopical changes at necropsy |  | - | | - |

^*^ All tests were performed or administrated by the Swedish National Veterinary Institute; IF= immunofluorescence. ^†^ Pathogen status of the sentinel rats housed in the barrier facility at the Scheele Laboratory, Karolinska Institutet from 2008-2013, which spans the entire period of the experiments in this study. ^‡^ Pathogen status of the conventional facility at Medical Inflammation Research at Lund University at approximately the same date as the experiment shown in figure 3A was made. ^4^ Pinworms.
