# Supplementary material for: Animal Models of Rheumatoid Arthritis (I): Pristane-Induced Arthritis in the Rat
Source: PLoS One. 2016 May 26;11(5):e0155936. doi: 10.1371/journal.pone.0155936 (PMC4881957; doi:10.1371/journal.pone.0155936)
Supplement: S1 Text — (DOCX) [file pone.0155936.s003.docx]

S1 Text

**BeTheCure Guidelines for Pristane-Induced Arthritis**

**Recommendations for disease induction, arthritis evaluation and reporting of data**

*Disease induction*: Animals should be anesthetized prior to the procedure using a mixture of isoflurane (1-3%) and oxygen (2-3 dm^3^/min). The rat should be placed on its abdomen with the tail pointing straight backwards. Seventy percent ethanol is applied to the tail base with a spray bottle whereafter the fur at the injection site should be parted along the midline. The needle should be inserted with the bevel facing upwards and clearly visible through the skin as demonstrated in Figure 2A (far right picture). A dose of 100 or 150 μl of synthetic pristane (See *Methods*) is injected strictly i.d., which can be distinguished from a s.c. injection by the difference in resistance. When the pristane has been injected, the thumb should be placed above the needle entry site, and the needle should be slowly withdrawn.

*Disease evaluation*: Depending on the age of the animals (Table 1), the first signs of arthritis will appear at day 9-14 after immunization (Figure 1B, left picture). The rats should be weighed on the first scoring day and thereafter at least two times per week. It is recommended to use a balance that offers a dynamic weighing mode, in particular if the animals cannot be fixed during weighing. The paws should be visually inspected at least twice a week, and preferably at the same time of the day, albeit a more frequent number of inspections may be necessary upon and shortly after onset of acute arthritis. Each paw receives a maximum of 15 points; 1 point is given for each inflamed knuckle or proximal interphalangeal (PIP) joint, whereas the ankle receives a score of 1-5, which should be proportional to the inflamed area (Figure S1). Scores are not given for deformations that are not accompanied by erythema (Figure S1G).

*Reporting of data*: Clinical disease data showing mean values and error bars (S.E.M.) should be reported. It is also highly recommended to include weight data, which should be depicted as percentage relative to the weight at disease onset – not to the day of immunization. In addition, sufficient information regarding number of arthritic animals, total number of animals, age-range, and substrain (including vendor) must be provided. Likewise, housing conditions (e.g. numbers and mixing in cages and enrichments) as well as alterations in the specific pathogen free (SPF) health status as recommended by the FELASA or AALAS should be specified along with the data. If treatment is included, it needs to be stated whether the different treatment groups were housed mixed or in separate cages. Since the scoring system is not quantitative, any statistical difference between mean arthritis scores should be determined using a non-parametric test. It is also highly recommended that the reporting of disease data follows the ARRIVE guidelines [49].
